# Will Smog Cause Mental Health Problems? Indication from a Microsurvey of 35 Major Cities in China

**DOI:** 10.3390/ijerph182312388

**Published:** 2021-11-25

**Authors:** Yanming Li, Ying Xin, Kangyin Lu, Wencui Du, Fei Guo

**Affiliations:** 1 School of Economics and Management, Northeast Normal University, Changchun 130117, China; liym001@nenu.edu.cn (Y.L.); luky440@nenu.edu.cn (K.L.); 2 School of Economics, Capital University of Economics and Business, Beijing 100070, China; xinying@cueb.edu.cn; 3 Information Center, Hebei Petroleum University of Technology, Chengde 067000, China; guof030@nenu.edu.cn

**Keywords:** air quality, mental health, ordered probit model

## Abstract

Using the survey data of 21,861 participants from 35 major cities in China in 2018 and 2019, the effect of air quality on participants’ mental health was empirically tested based on the ordered probit model. The results showed that smog can significantly influence the mental health of participants. The better the air quality, the better the participants’ mental health, while poor air quality results in poor mental health. The older and higher-paid participants demonstrated poorer mental health. Additionally, for different health conditions, the air quality had different effects on the participants’ mental health. The healthier the participants, the more sensitive their mental health to changes in air pollution; the poorer the physical condition of the participants, the less sensitive their mental health to changes in air quality. Therefore, we need to more comprehensively and scientifically understand the effect of air quality on health. We need to pay attention not only to the adverse effects of smog on participants’ physical health, but also to its effects on participants’ mental health to improve both the physical and mental health of participants by improving the air quality.

## 1. Introduction

The level of economic and social development of developing countries, represented by China, has been constantly increasing alongside rapid industrialization and urbanization. Simultaneously, air quality issues are becoming increasingly severe. According to the 2020 Environmental Performance Index (EPI), jointly released by the Yale Center for Environment Law and Policy and other institutions, China ranked 120 out of the 180 countries in 2020 with an environmental performance index of 37.3, a decrease of 13.44 points from 2019, and its comprehensive environmental performance was poor [1]. The 2019 China Ecological Environmental Conditions Bulletin showed that the ambient air quality of 180 cities did not meet the stipulated standard, accounting for 53.4% of the total of 337 cities at the prefecture level and above in China [2]. These 337 cities experienced hazardous pollution for 452 days and serious pollution for 1666 days, accumulatively an increase of 88 days from 2018. The number of days when PM_2.5_, PM_10_, and O_3_ were major pollutants accounted for 78.8%, 19.8%, and 2.0%, respectively, of the days categorized as serious pollution or above, indicating a continued severe air pollution situation. The leading cause of air pollution remains the excessive concentration of inhalable particulates.

The World Health Organization’s definition of health is a state of complete physical, mental, and social well-being, and not merely the absence of disease or infirmity [3]. Mental health problems have now become one of the most important public health problems in developing countries. According to the White Paper on Mental Health of Chinese Urban Residents, in 2018, 73.6% of the participants in China were in mental subhealth condition, and 16.1% reported having different degrees of psychological problems; the mentally healthy participants only accounted for 10.3% of the total. Over 80% of the people with mental health problems, including those with neurological disorders and drug abusers, have little access to high-quality and affordable mental health care. Mental health is the core content of the connotation construction of health management, an important part of health management disciplinary construction, and an aspect of concern in overall global health. The ability to improve residents’ mental health is related to the healthy development of residents, and it is an important factor that determines the sustainable development of a city and a nation.

To improve residents’ mental health, the first task is to clarify how air quality influences their mental health. First, the human brain is sensitive to light owing to the presence of the pineal gland. Cells are less active when the gland perceives enough light. However, it becomes active and inhibits the production of some hormones in the human body when the external environment changes, such as when it becomes dark or when it is unable to be exposed to sunlight. Among the suppressed hormones, thyroxine and adrenaline wake up the human body [4]. Hence, strong sunlight positively influences people’s mood, making them more optimistic. Conversely, poor sunlight and smog make people feel depressed and dispirited, further exacerbating depression, leading to an increased suicide rate. Second, air pollution influences residents’ physical health, which, in turn, can cause mental health problems. The human body has pathological responses to excessive sulfides and carbon dioxide concentrations in the air, such as respiratory diseases or a mental decline [5,6]. Additionally, when air pollution causes physical health problems, patients are unable to perform the same daily routines as healthy people, resulting in the emotions of repulsion and antipathy, which may result in psychological burden and mental health fluctuations due to the cost of medical care. In conclusion, air quality mainly affects mental health through its direct influence on the nervous system and its indirect influence on physical health.

This study, from the perspective of air quality, adopted the survey data of 21,861 participants from 35 major cities in China between 2018 and 2019 and, based on the ordered probit model, empirically tested the effect of urban air quality on participants’ mental health and the role of physical health in the above relationship, in order to provide policy suggestions for developing countries to further boost participants’ mental health and achieve the goals of improving the quality of life and air. The rest of this paper is organized as follows: Section 2 provides a summary and evaluation of the literature on the relationships between the factors influencing air quality and mental health; Section 3 provides an analysis of the mental health of the 21,861 participants from 35 major cities in China and the air quality of each city; Section 4 presents the measurement model and definitions of the key variables and specifies the data sources to empirically test the effect of air quality on mental health; and Section 5 summarizes the main findings of this study and provides policy suggestions for improving the mental health of participants from the perspective of air quality.

## 2. Literature Review

### 2.1. Studies on the Factors Influencing Mental Health

Researchers have classified the factors that influence mental health into two categories: individual factors, including the feeling of being left behind [7], parent–child relationships [8], academic stress [9,10], work stress [11,12,13,14], and physical health [15,16]; and environmental factors, including social support [17] and profession [18,19,20]. (1) A poor parent–child relationship will have a negative impact on children’s mental health. For example, Wang (2019) found that the mental health of left-behind children significantly correlates with the pre- and post-migration of parents, manifesting as emotional and behavioral disorders and hyperactivity [7]. Nancy et al. (2018) revealed that parents with PTSD (post-traumatic stress disorder) and depression negatively affect their children [8]. In particular, the effects on girls are more prominent than on boys, including experiencing humiliation, external behaviors (fighting, using bad language, and having no respect for people), and missing school. (2) Academic stress is not conducive to students’ physical and mental health. For example, Cosma et al. (2020), based on a transnational empirical study, found that the effect of academic stress on students’ mental health increases over time. In particular, high academic stress aggravates the psychosomatic illnesses of girls and overage teenagers [10]. (3) Work stress is not conducive to students’ physical and mental health. For example, Virtanen et al. (2007) found that the correlation between work stress and depression or anxiety disorder in the workplace is more prominent in men than in women, and the use of antidepressants will only be pronounced in men in the future [14]. (4) Physical health is also conducive to mental health. For example, Cohen et al. (2007) reported that the elderly who often participate in art activities enjoy better physical health, which has a strong positive effect on their mental health [16]. (5) Social care will promote mental health. For example, Moshe and Gerald (2016) found that social support can be an effective intervening variable of the emotional quotient that influences mental health [17]. Effective emotion regulation can promote social interaction, and good social interaction will enhance psychological well-being. (6) Some industries have special mental health problems. For example, Rathod et al. (2018) conducted a study from the perspectives of workplace exposure, promotion pressure, and customer group, and found that people in the service industry, such as the leather, chemical, oil paint, catering, and urban public transport service sectors in Canada, are at higher risk of mental health problems [20].

### 2.2. Studies on Air Quality and Physical Health

Studies on the relationship between air quality and health mainly focus on the effects of air quality on physical health. Some studies have found that air pollution can cause respiratory diseases [21,22]. Infants and young children, being in the developmental stage, are vulnerable to respiratory tract infections and bronchitis, as their respiratory defense mechanisms are different from those of adults; thus, air pollutants increase their susceptibility to disease. Additionally, the combination of air pollutants with airborne pollen and fungal propagules enhances the release of antigens, which can cause allergic symptoms and lead to acute asthma when inhaled [21]. An increasing number of studies have demonstrated that air quality may increase the risk of cardiovascular disease [23,24]. Long-term exposure to high concentrations of inhalable PM_10_ is one of the main causes of death of patients with cardiovascular diseases, especially with ischemic heart disease [25]. Air pollution may also lead to high stroke mortality. Stroke mainly occurs in patients with cardiovascular diseases and obese patients, who may suffer from systematic inflammation and autonomic dysfunction when inhaling polluted air, thus leading to a stroke [26]. Some studies have suggested that the years of life lost (YLL) may increase as a result of the premature mortality of patients with the growing concentration of air pollutants such as PM_2.5_, PM_10_, SO_2_, and NO_2_ [27], and the increase in PM_2.5_ concentration may lower the average life expectancy [24].

### 2.3. Studies on Air Quality and Mental Health

Generally, the research on the effects of air quality on mentality is relatively detailed. Since 1964, a number of papers have included the indexes of specific pollutants in the analysis of air quality. For instance, it has been found that excessive mercury in the air causes toxicological damage to the nervous system of the brain [28]. A study, based on the data from the Baltimore Schizophrenia Emergency Clinic in Maryland, U.S., found that the monthly number of inpatients with mental disorders is significantly correlated with the air quality in Baltimore, and unexplained schizophrenia, drug-induced psychosis, and alcohol-induced psychosis are significantly correlated to PM_2.5_ [29]. Some Chinese scholars have conducted a time series analysis of the short-term correlation between the incidence of common diseases (psychosis and non-accident-related) and atmospheric pollutants in Tianjin between 2008 and 2011, finding that psychosis incidence (length of stay in hospital due to psychosis) increases 0.06%, 0.1%, and 0.1% as a result of an increase of 10 μg/m^3^ in the PM_10_, SO_2_, and NO_2_ concentration, respectively, every two days [30]. Other scholars have evaluated the potential relationship between car-produced pollutants (benzene, CO, and NO_2_) and the risk of schizophrenia, and the role of the concentration of airborne solid suspended particulate matter in psychosis incidence [31,32]. However, few of these studies have directly discussed the effects of air quality on residents’ mental health; only a few have indirectly analyzed the effects of environment quality on residents’ mental health from the perspectives of exposure to a green environment [33] and traffic noise pollution [34]. For example, Gu et al. (2015), based on the 2014 China Migrants Dynamic Survey (CMDS), found that atmospheric pollution remarkably increases people’s mental block using the ordered probit method [35]. By controlling the social demographic variables, Tao et al. (2015) used the probit model to empirically analyze the effects of air pollution on the elderly’s mental health in China in 2015, and found that sulfur dioxide emissions have a strong influence on the mental health of the elderly, showing a U-shaped curve [36]. Sui et al. (2018), based on the 2017 Questionnaire Survey of Female College Students in Anshan City, China, and the Cornell Medical Index (CMI) and General Well-Being (GWB) schedule, used a generalized linear mixed model for an empirical study, and found that exposure to ambient air pollution (mainly PM_2.5_) may be a major contributor to mental health problems among female college students [37].

## 3. Methodology

### 3.1. Measurement Model and Variable Definition

In order to test the effect of air quality on participants’ mental health, the following measurement model was designed based on existing research [36,38] and data availability.

(1) $$health_{\mathrm{it}}=\alpha_{0}AQI_{it}+\alpha_{1}age_{it}+\alpha_{2}gender_{it}+\alpha_{3}edu_{it}+\alpha_{4}income_{it}+\alpha_{5}workhour_{it}+\alpha_{6}edu\_child_{it}+\varepsilon_{it}$$

where participants’ mental health (*health*) is a dependent variable; air quality (*AQI*) is an independent variable; age (*age*), gender (*gender*), educational level (*edu*), income (*income*), daily work hours (*workhour*), and children’s education (*edu_child*) are control variables; ε is a stochastic disturbance term; *i* denotes individual participant; and *t* stands for year.

**Figure 1:**
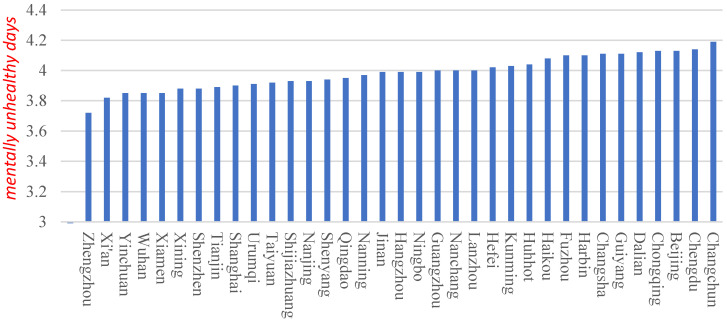
Mental ill-health condition of the participants in 35 major cities.

Participants’ mental health (*health*) is a dependent variable, expressed as the number of days free from anxiety, depression, or emotional incontinence (mentally healthy) in the past 30 days of participants in this paper. If the participants experienced less than five mentally healthy days, they were considered to be in the worst mental health, assigned a value of 1; if the participants experienced 6–11 mentally healthy days, they were considered to have worse mental health, assigned a value of 2; if the participants experienced 12–17 mentally healthy days, they were considered to have average mental health, assigned a value of 3; if the participants experienced 18–23 mentally healthy days, they were considered to have good mental health, assigned a value of 4; and if the participants experienced 24–30 mentally healthy days, they were considered to have the best mental health, assigned a value of 5. Figure 1 presents the statistics of the mental health status of the participants in 2018 and 2019, and most of them had good mental health. In order to measure the effect of air quality on mental health, air quality was introduced into the model to examine the change in the participants’ mental health status.

The higher the AQI value, the worse the air quality. According to the AQI value, there are six levels of air quality: good, satisfactory, moderately polluted, poor, very poor, and severe. Given the possible time lag of the effect of air quality on the participants’ mental health, the air quality index of these cities of 2017 and 2018 was used for testing.

In order to minimize the error of estimation due to omitted variables, other individual characteristics were introduced that may influence participants’ mental health as control variables [39]: age (*age*), gender (*gender*), educational level (*edu*), income (*income*), daily work hours (*workhour*), and children’s education (*edu_child*). The analysis is presented below.

Age (*age*): The participants might have had different mental statuses, as they were born in different eras, experienced a different stage of economic and social development, and had different tolerances to air quality. For example, some older participants might have mental health problems owing to life stress and poor physical health. Additionally, some participants born before the reform and opening-up might be struggling to adapt to the rapid development of industrialization and urbanization. They clearly felt the large changes in society, but their mental status has not yet adapted to the rapid development of the era, thus making them prone to mental ill-health. Therefore, the age bracket of participants was assigned a value. If the participant was in the age bracket of 20–29 years, a value of 1 was assigned; if the participant was in the age bracket of 30–39 years, a value of 2 was assigned; if the participant was in the age bracket of 40–49 years, a value of 3 was assigned; if the participant was in the age bracket of 50–59 years, a value of 4 was assigned; and if the participant was aged 60 years or above, a value of 5 was assigned.

Gender (*gender*): If the participant was a man, a value of 0 was assigned; if the participant was a woman, a value of 1 was assigned. The participants were of different sexes, and mental health status may follow the rule of sex differences. Women experience higher rates of emotional and anxiety disorders than men owing to their physiology and their perception of the outside world. Women are more sensitive than men and are more prone to fluctuations in psychological status [40].

Educational level (*edu*): In general, people with a lower educational level are prone to psychological imbalances and even extreme thoughts under the weight of survival stress and employment pressure. However, the higher the educational level, the higher the self-cognition ability. Thus, it was considered that participants with higher psychological endurance and self-regulation ability would have a better level of mental health than those with a lower educational level. Therefore, the highest educational level of the participants was assigned a value. If the participant was uneducated, a value of 1 was assigned; if the participant’s highest educational level was elementary school, a value of 2 was assigned; if the participant’s highest educational level was high school (including secondary vocational school), a value of 3 was assigned; if the participant’s highest educational level was a Bachelor’s degree, a value of 4 was assigned; and if the participant’s highest educational level was a Master’s degree or above, a value of 5 was assigned.

Income (*income*): A participants’ income not only determines their financial situation, but likely influences their mental health. Generally, the higher a participants’ income, the more optimistic they are about the future, the less mentally stressed they are than lower-paid participants, the better their mental status, and the mentally healthier they are. In this study, the income level of the participant was assigned a value. If the participant’s income was less than RMB 2000, a value of 1 was assigned; if the participant’s income was RMB 2001–5000, a value of 2 was assigned; if the participant’s income was RMB 5001–8000, a value of 3 was assigned; if the participant’s income was RMB 8001–15,000, a value of 4 was assigned; and if the participant’s income was greater than RMB 15,001, a value of 5 was assigned.

Daily work hours (*workhour*): Daily work hours influence both the physical and mental health of participants. The longer the work hours, the more physically and mentally exhausted they are. When exhausted, they are more likely to have negative emotions, which influences their mental health. In this study, the daily work hours of the participant were assigned a value. If the participant had no job, a value of 1 was assigned; if the participant’s work hours were no more than 4 h, a value of 2 was assigned; if the participant’s work hours were 4–8 h, a value of 3 was assigned; if the participant’s work hours were 8–10 h, a value of 4 was assigned; and if the participant’s work hours were more than 10 h, a value of 5 was assigned.

Children’s education (*edu_child*): The participants whose children were enrolled in ordinary high and elementary school likely paid more attention to their children. Too much attention on and input in children’s studies would have placed pressure on the participants. Hence, the participants whose children were in compulsory education could have suffered from greater mental stress than those without children in ordinary high or elementary school. Therefore, if the participant’s children were in ordinary high or elementary school, a value of 1 was assigned; otherwise, the value was 0.

### 3.2. Model Selection

The ordered probit model was used to estimate the measurement model as the dependent variable, participants’ mental health (*health*), is a discrete variable, not a continuous variable. Participants’ mental health (*health*) represents the participants’ mental health on a scale of 1 to 5. *health* = 5 indicates the healthiest, and *health* = 1 indicates the least healthy. According to Wooldridge (2001) [41], an ordered probit model of *y* can be derived from a latent variable model. Assume that the latent variable is determined by the following equation:

(2) $$y^{*}=x\beta +e\;\;\;\;\;e|x∼Normal\left(0,1\right)$$

where *β* represents the vector K × 1, assuming that $\alpha_{1}<\alpha_{2}<\cdots <\alpha_{5}$ represents the unknown cut point; then, the probability of each response can be calculated as follows:

(3) $$P\left(y=1|x\right)=P\left(y^{*}\leq \alpha_{1}|x\right)=P\left(x\beta +e\leq \alpha_{1}|x\right)=\Phi \left(\alpha_{1}-x\beta \right)$$

(4) $$P\left(y=2|x\right)=P\left(\alpha_{1}\leq y^{*}\leq \alpha_{2}|x\right)=\Phi \left(\alpha_{2}-x\beta \right)-\Phi \left(\alpha_{1}-x\beta \right)$$

(5) $$P\left(y=3|x\right)=P\left(\alpha_{2}\leq y^{*}\leq \alpha_{3}|x\right)=\Phi \left(\alpha_{3}-x\beta \right)-\Phi \left(\alpha_{2}-x\beta \right)$$

(6) $$P\left(y=4|x\right)=P\left(\alpha_{3}\leq y^{*}\leq \alpha_{4}|x\right)=\Phi \left(\alpha_{4}-x\beta \right)-\Phi \left(\alpha_{3}-x\beta \right)$$

(7) $$P\left(y=5|x\right)=P\left(y^{*}>\alpha_{5}|x\right)=1-\Phi \left(\alpha_{4}-x\beta \right)$$

The maximum likelihood method is used to estimate the above model to obtain the estimated value.

### 3.3. Data Sources

The data used in this paper come from two parts: one is the survey data of 35 cities, which is the unique data of this study; the other is the AQI (air quality index) data of 35 cities, which comes from the daily air quality index of 35 cities published by National Air Quality Real-Time Platform of the Ministry of Ecology and Environment of China, and calculates the average value of AQI index of the city in a year, representing the city’s air quality in a year. This study surveyed 21,861 participants from 35 major cities in China in 2018 and 2019, and the data were obtained from the Report on Quality of Life in Chinese Cities jointly released by the Institute of Economics of Chinese Academy of Social Science and the School of Economics of Capital University of Economics and Business in 2018 and 2019. The survey has lasted for 10 years since 2011. After years of a complete evaluation index system, the survey results are relatively stable. The data were collected by administering a questionnaire over the telephone and Internet to the participants of 35 major cities in 30 provinces in China, which is still representative. A total sample size of 21,861 was polled, consisting of 12,878 in 2018 and 8983 in 2019. The sampling distribution is shown in Table 1.

### 3.4. A Comparison of the Current Air Quality in the Big Cities of China

**Figure 2:**
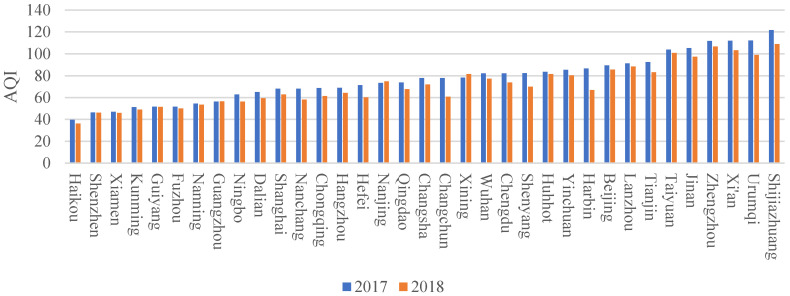
Air quality of the 35 major cities of China in 2017–2018.

In this study, the annual average air quality index (AQI) was used to measure the air quality of China’s major cities. This index includes the concentrations of six major pollutants: fine particulate matter, inhalable particulate, sulfur dioxide, nitrogen dioxide, ozone, and carbon monoxide. It is a calculated composite index, ranging from 0 to 500; the higher the AQI value, the worse the air quality. The trend in the AQI of China’s 35 major cities between 2017 and 2018 is shown in Figure 2. In 2017, the annual average AQIs of Haikou, Xiamen, Shenzhen, and Kunming were 36.31, 46.00, 46.12, and 48.99, respectively, each with an air quality grade of “good”. These cities had the best air quality of the 35 investigated cities. The top 10 cities in terms of air quality in 2017 were Haikou, Shenzhen, Xiamen, Kunming, Guiyang, Fuzhou, Nanning, Guangzhou, Ningbo, and Dalian. The average AQI value ranged from 40 to 60. By comparison, the top 10 cities for air quality in 2018 did not change considerably from 2017, and there was no significant difference between individuals. The last 10 cities in terms of air quality in 2017 were Harbin, Beijing, Lanzhou, Tianjin, Taiyuan, Jinan, Zhengzhou, Xi’an, Urumqi, and Shijiazhuang, with their average AQI value ranging between 95 and 110. The air quality ranking of the last 10 cities in 2018 changed a little, with no significant fluctuations between individuals over the two years, except that the AQI of Harbin was 86.7 in 2017, but 67 in 2018.

### 3.5. A Comparison of the Current Mental Health of the Participants in the Big Cities of China

In this study, the Center for Epidemiological Studies—Depression Scale (CES-D) was used to measure the mental health of participants based on the number of days and frequency of mental health issues. Of these participants, 4872 (~22.20% of the total) did not experience anxiety, depression, or emotional issues (“not very mentally healthy”) on a single day (checked “zero days”), indicating that most participants recently had good mental health. Meanwhile, 284 participants (~1.30% of the total) reported experiencing those problems every day. This low proportion reveals that the mental health of this small number of participants was poor, and so more attention should be paid to their mental health and address corresponding mental health problems. There were 11,217 participants (~51.31% of the total) who checked the option of “less than 12 days”, i.e., they were mentally unhealthy for less than three days per week, indicating that a large number of participants experienced problems with mental ill-health, but were generally in good mental health. Their mental health could likely be improved if they address it seriously as early as possible, and the number of mentally unhealthy days might be reduced to 0 within 30 days.

Figure 1 presents the mental health status of the samples from the surveyed cities in 2018 and 2019. It shows that the number of mentally unhealthy days of participants in these 35 major cities ranged between 3.8 and 4.2, indicating that most participants had good mental health. Of these 35 sample cities, Zhengzhou participants had good mental status, with an average score of 3.7, while Changchun participants had a poor mental status, with an average score of 4.2. The participants in the following 21 cities had 3.8–4 mentally unhealthy days: Xi’an, Yinchuan, Wuhan, Xiamen, Xining, Shenzhen, Tianjin, Shanghai, Urumqi, Taiyuan, Shijiazhuang, Nanjing, Shenyang, Qingdao, Nanning, Jinan, Hangzhou, Ningbo, Guangzhou, Nanchang, and Lanzhou, ranked from low to high, accounting for 60% of the 35 cities. This suggests that the participants in these cities overall had 3.8–4 mentally unhealthy days in 2018–2019, which falls within a reasonable range. In the 13 cities represented by Changchun, the participants had more than four mentally unhealthy days. These cities were as follows: Hefei, Kunming, Haikou, Fuzhou, Harbin, Changsha, Guiyang, Dalian, Chongqing, Beijing, and Chengdu, ranked from low to high. These cities are geographically diverse and the inter-provincial effects, such as economic development level and demographic factors, may have had no direct correlation with the participants’ mental health.

## 4. Results Analysis

### 4.1. Descriptive Statistics

Table 2 presents the descriptive statistics of the key variables. The descriptive statistics of the full range of samples showed little individual difference in the participants’ mental health (*health*), with a mean value of 4.69, indicating that the participants were free from anxiety, depression, and emotional issues (“not very mentally healthy”) for nearly more than three weeks within 30 days. The sample mean was 4.77 in 2018 and 4.57 in 2019, which shows that the mental health of the participants in 2018 and 2019 was good. However, the mental health of the participants in 2018 was better than in 2019. The standard deviation in the participants’ mental health reveals that the 2019 samples were more discrete than the 2018 samples, but with little fluctuation in general. Most of the participants were in the age bracket of 20–29 years, indicating that the majority of them were young people. In terms of the sex of the participants, there was an equal percentage of women and men. The educational level of the full-range samples scored 3.72, and the educational level of the 2018 and 2019 samples scored 3.57 and 3.92, respectively, indicating that most of the participants had a Bachelor’s degree. Regardless of the full-range sample mean of the income level and the sub-sample means of 2018 and 2019, the monthly income of the participants ranged from RMB 5000 to 8000, and they usually worked 4–8 h every day. Finally, the proportions of participants who had children in school or not showed that the samples with no children in elementary or high school accounted for a larger proportion.

### 4.2. Basic Regression

The ordered probit model was used to estimate the measurement model with the mixed cross-sectional data of 2018–2019 as samples to test the effect of air quality on the participants’ mental health. The regression results are shown in Table 3. The heteroskedasticity in Column (1) in Table 3 is not uncorrected; Column (2) presents the estimated results after the heteroskedasticity was corrected. In order to test the credibility of the regression results, a robustness test was performed based on the model and data availability. The report results are shown in Columns (3)–(6).

In Table 3, the regression in Columns (1) and (2) shows that the estimated coefficient of the air quality index (*AQI*) was significantly negative at the level of 1%, which means that, the poorer the air quality, the higher the AQI, and the fewer the number of mentally healthy days of the participants. The estimated result reveals that the air quality notably lowered the mental health level of the participants, becoming an important factor influencing participants’ mental health. This conclusion is still tenable after considering the problem of heteroskedasticity. The regression results of the control variable also provide some information. The regression coefficients of age (*age*) were significantly negative in Columns (1) and (2), which shows that an inverse relationship exists between the mental health and the age of the participants. The older the participant, the fewer the number of mentally healthy days. This is in line with the findings of a previous empirical study (Tao et al., 2015). The regression coefficients of gender (*gender*) in Columns (1) and (2) were remarkably positive, indicating a greater fluctuation in mental health in female participants than in male participants. The estimated coefficients of educational level (*edu*) were remarkably positive in the regression in Columns (1) and (2), indicating that, the higher the participants’ education level, the better their mental health. The estimated coefficients of income (*income*) were remarkably negative in the regression in Columns (1) and (2), indicating that, the higher the participants’ income, the fewer the number of mentally healthy days. The regression coefficients of daily work hours (*workhour*) were remarkably positive in the regression in Columns (1) and (2), indicating that, the longer the working hours, the better the mental health compared with the participants working fewer hours. The estimated coefficients of children’s education (*edu_child*) were remarkably negative in the regression in Columns (1) and (2), indicating a negative correlation between whether the participants’ children were in ordinary elementary and high school and the mental health of participants. The participants whose children were in elementary and high school reported fewer mentally healthy days than those with no children in elementary or high school.

The main methods used for the robustness test in this study were time-phased regression and change in sample range. Time-phased regression was used to divide the samples into a 2018 group and a 2019 group for basic regression. The results are shown in Columns (3) and (4) in Table 3. The results show that, in the regression of these two groups, the estimated coefficient of the air quality index (*AQI*) was significantly negative at the level of 1%, which means that, in 2018 and 2019, the poorer the air quality, the higher the number of mentally healthy days. Air quality issues such as smog can remarkably lower the mental health level of participants, and the regression results are robust.

The second method involved changing the sample range. Given the fast pace of life and the extreme life stress in megacities, the mental health of some participants could have been largely influenced by the mental stress caused by rapid city development. Therefore, the remaining 19,172 samples were used for re-regression in terms of the 2689 participants from Beijing, Shanghai, and Guangzhou. The results are shown in Column (5) in Table 3. The results show that the estimated coefficients of the air quality index (*AQI*) were still significantly negative, and the coefficients, either positive or negative, remained unchanged. Thus, the conclusion that a worsening air quality is harmful to participants’ mental health remains unchanged.

Finally, the key variables were replaced. The air quality compliance rate was considered as a substitution for the urban air quality index. The specific calculation method involved determining the percentage of days with an annual air quality index greater than 100 in these 35 cities. The higher the compliance rate, the better the air quality. The regression results are shown in Column (6) in Table 3. The results show that the estimated coefficients of the air quality index (*AQI*) were still significantly positive, which indicates that the air quality compliance rate and the number of mentally healthy days are positively correlated, and that improved air quality is conducive to enhancing participants’ mental health. This proves that the above conclusion is still robust.

### 4.3. Further Testing Based on Physical Health

In addition to repression, anxiety, and the effects on mental health, air quality can influence people’s physical health. Studies have found that a worsening air quality can cause respiratory diseases and other physical health problems [42]. When people’s physical health is influenced, they may feel down and anxious, and then become mentally unhealthy. In the 2018–2019 questionnaires, there were two questions about physical health: “How do you feel about your health condition in general? and “How long did you get ill or injured (physically unhealthy) in the past 30 days?” According to the answers to the first question, the participants were divided into a group of physically healthy participants (18,480 participants, accounting for 84.5%) and a group of physically unhealthy participants (3381 participants, accounting for 15.5%) to repeat the basic regression separately. The results are shown in Columns (1) and (2) in Table 4. Then, according to the answers to the second question, we calculated the average number of physically unhealthy days of the samples and divided them into a group of physically unhealthy participants and a group of physically healthy participants to repeat the basic regression. The results are shown in Columns (3) and (4) in Table 4. Thus, the regression in Columns (1) and (3) shows that the estimated coefficients of the air quality index (*AQI*) were significantly negative, which indicates that a worsening air quality can increase anxiety, nervousness, and depression for physically healthier participants, thus leading to mental health problems. This is consistent with the results of the basic regression and the robustness test. However, according to the regression result of item (2), the estimated coefficients of the air quality index (*AQI*) were significantly positive; the regression results of item (4) show that the estimated coefficient was non-significant, indicating that, for physically unhealthy participants, a worsening air quality does not result in increasing mental health problems; when the participants suffer from illness, they have no time to spare for air quality and mental health problems.

## 5. Conclusions

With the 21,861 valid samples obtained from the 2018–2019 Quality of Life Index of Chinese Cities as the objects of study, the effects of air quality on participants’ mental health using the ordered probit model were empirically investigated in this study. The conclusions are as follows. First, a positive correlation was found between air quality and participants’ mental health by creating a regression model. The better the air quality, the more mentally healthy days. Thus, it was found that improved air quality can significantly and positively enhance participants’ mental health, and vice versa. Second, factors such as age, income, and children’s education also have a certain effect on the mental health of participants. The mental health status of participants was shown to worsen with age—the higher the participant’s income, the fewer their number of mentally healthy days—and the influence of whether their children are in school cannot be neglected. Third, a further analysis on participants in different physical health conditions revealed that the participants who are less physically healthy as a result of air pollution are more likely to suffer from more mental health problems due to changes in air quality.

## 6. Suggestions

Although many countries are now stressing the importance of sustainable development, and attention has been paid to environmental pollution and improvement, it is a step-by-step process to shift the emphasis from the effect of environmental pollution on ecology and economy to the effect on individual physical and mental health. Scientific studies and empirical testing can be used to construct a framework for sustainable ecological development in the future. Improving air quality is still a hot topic; a negative effect of smog on people’s mental health was found. Current actions have resulted in some improvements in air quality, but further action is needed. Currently, the main cause of smog in China is the increase in secondary aerosol concentration in the air as a result of the increase in carbon dioxide generated by coal combustion and the emissions of sulfur dioxide and nitric oxide from thermal power plants. Therefore, further efforts should be directed toward emission reduction and ensuring emissions meet the standards. China’s current advances in clean technology and the introduction of strict standards by the government have contributed to this goal. In the future, China must continue the path of green emission reduction to ensure the sustainable improvement in air quality.

Green emission reduction is not only about supervising and urging the government to attach more importance to reductions in PM_2.5_ emissions and inhalable particulate concentrations and strengthening environmental protection supervision, but also about urging the government to provide more help to those whose mental illness is aggravated by air pollution in the spirit of humanity. The WTO study data showed that more than three-quarters of people suffer from mental illnesses, but they have no access to effective medical or healthcare services, which means that a gap remains in mental healthcare. Regardless of the empirical conclusion drawn based on China’s data or the findings of scholars in the rest of the world, participants’ mental health must be improved:

(1) Life tips: The health department should repeatedly remind people to wear masks outside when the inhalable particulate concentration is higher than standard, and encourage them to participate in outdoor activities on sunny days. Although these two tips are common knowledge, they are often ignored by people.
(2) Community services: A free mental health inquiry service should be provided in the community so that patients can be identified and seek medical advice as soon as possible.
(3) Medical support: For patients whose illness is aggravated as a result of air pollution, the government should bear their medical costs or provide subsidies to alleviate their expense burdens.
(4) Site support: Both mental and physical health require individuals to have better self-regulation ability. The overall health of society will be enhanced if more public places are open (as appropriate). Participants who are physically and mentally healthy considerably benefit the sustainable development of human society. All countries around the world should work together to actively engage in mental health construction.

## Figures and Tables

**Table 1:** Sample distribution.

City	2018	2019	Total	City	2018	2019	Total
Beijing	655	388	1043	Chengdu	343	235	578
Shanghai	521	387	908	Kunming	336	235	571
Urumqi	332	234	566	Hangzhou	335	233	568
Lanzhou	332	235	567	Wuhan	437	310	747
Nanjing	425	311	736	Shenyang	336	234	570
Nanning	339	234	573	Jinan	340	235	575
Nanchang	331	236	567	Haikou	339	232	571
Xiamen	334	237	571	Shenzhen	433	311	744
Hefei	351	235	586	Shijiazhuang	337	232	569
Hohhot	343	235	578	Fuzhou	332	234	566
Harbin	342	238	580	Xining	339	235	574
Dalian	337	234	571	Xi’an	325	235	560
Tianjin	448	315	763	Guiyang	336	233	569
Taiyuan	334	234	568	Zhengzhou	332	232	564
Ningbo	339	234	573	Chongqing	433	316	749
Guangzhou	408	316	724	Yinchuan	344	234	578
Changchun	361	235	596	Qingdao	333	235	568
Changsha	336	234	570			Total	21,861

Note: These 35 cities include four municipalities directly under the Chinese central government (Beijing, Shanghai, Tianjin, and Chongqing), 16 provincial capitals (Urumqi, Lanzhou, Nanning, Nanchang, Hefei, Hohhot, Taiyuan, Changsha, Kunming, Haikou, Shijiazhuang, Fuzhou, Xining, Guiyang, Zhengzhou, and Yinchuan), five sub-provincial cities (Xiamen, Dalian, Ningbo, Shenzhen, and Qingdao), and 10 cities that are both provincial capitals and sub-provincial cities (Nanjing, Harbin, Guangzhou, Changchun, Chengdu, Hangzhou, Wuhan, Shenyang, Jinan, and Xi’an).

**Table 2:** Descriptive statistics of the key variables.

Variable	Sample Size	Mean Value	Standard Deviation	Minimum Value	Maximum Value
Panel A: Full-range samples					
health	21,861	4.69	0.81	1	5
AQI	21,861	74.50	19.63	36.31	121.7
age	21,861	1.80	1.02	1	5
gender	21,861	0.53	0.50	0	1
edu	21,861	3.72	0.76	1	5
income	21,861	2.60	1.13	1	5
workhour	21,861	3.05	1.16	1	5
edu_child	21,861	0.40	0.49	0	1
Panel B: Samples of 2018					
health	12,879	4.77	0.71	1	5
AQI	12,879	76.9	20.12	39.7	121.7
age	12,879	1.94	1.11	1	5
gender	12,879	0.49	0.5	0	1
edu	12,879	3.57	0.86	1	5
income	12,879	2.50	1.12	1	5
workhour	12,879	2.99	1.21	1	5
edu_child	12,879	0.39	0.49	0	1
Panel C: Samples of 2019					
health	8982	4.57	0.91	1	5
AQI	8982	71.06	18.38	36.31	108.93
age	8982	1.6	0.83	1	5
gender	8982	0.59	0.49	0	1
edu	8982	3.92	0.53	1	5
income	8982	2.73	1.12	1	5
workhour	8982	3.15	1.10	1	5
edu_child	8982	0.41	0.49	0	1

**Table 3:** Basic regression and robustness test.

	−1	−2	−3	−4	−5	−6
			Samples of 2018	Samples of 2019	Megacities Excluded	Substitution Variable
AQI	−0.093 ***	−0.341 ***	−0.109 ***	−0.149 ***	−0.086 ***	0.002 ***
	(−3.54)	(−2.09)	(−3.31)	(−3.55)	(−3.20)	−3.19
*age*	−0.160 ***	−0.129 ***	−0.135 ***	−0.110 ***	−0.167 ***	−0.163 ***
	(−19.42)	(−2.71)	(−13.27)	(−7.17)	(−19.97)	(−19.18)
*gender*	0.220 ***	0.185 ***	0.218 ***	0.144 ***	0.205 ***	0.213 ***
	−15.1	−2.15	−11.13	−6.31	−13.16	−13.68
*edu*	0.232 ***	0.381 ***	0.196 ***	0.048 ***	0.236 ***	0.232 ***
	−20.51	−4.19	−15.35	−1.89	−21.12	−20.32
*income*	−0.054 ***	−0.091 ***	−0.096 ***	−0.033 ***	−0.082 *	−0.062 ***
	(−6.59)	(−2.00)	(−8.55)	(−2.56)	(−10.03)	(−7.72)
*workhour*	0.097 ***	0.193 ***	0.071 ***	0.109 ***	0.078 ***	0.072 ***
	−10.68	−3.47	−6.17	−7.26	−10.66	−9.66
*edu_child*	−0.089 ***	−0.098 ***	−0.111 ***	−0.026 ***	0.094 ***	−0.070 ***
	(−5.76)	(−1.15)	(−5.37)	(−1.05)	−5.6	(−4.11)
Year	Controlled	Controlled	Uncontrolled	Uncontrolled	Controlled	Controlled
City	Controlled	Controlled	Controlled	Controlled	Controlled	Controlled
Pseudo-*R*^2^	0.018	0.222	0.118	0.069	0.109	0.104
Sample size	21,861	21,861	12,879	8982	19,172	21,861

Note: The number in brackets is the *t*-value; * and *** indicate significance at the 0.1 and 0.01 levels of the estimated coefficients, respectively, and the estimated result of the constant term is omitted.

**Table 4:** Further testing based on physical health.

	−1	−2	−3	−4
	Physically Healthy Group	Physically Unhealthy Group	Physically Healthy Group	Physically Unhealthy Group
AQI	−0.002 ***	0.038 ***	−0.146 ***	−0.011
	(−3.17)	−4.76	(−4.57)	(−0.20)
*age*	−0.229 ***	−0.310 ***	−0.121 ***	0.154 ***
	(−7.73)	(−3.90)	(−11.40)	−10.35
*gender*	0.441 ***	0.670 ***	0.210 ***	−0.02
	−5.46	−5.75	−11.77	(−0.83)
*edu*	0.326 ***	0.582 ***	0.241 ***	−0.033
	−8.64	−7.32	−17.17	(−1.13)
*income*	−0.389 ***	−0.514 ***	−0.054 ***	0.028
	(−4.93)	(−9.50)	(−5.39)	−1.84
*workhour*	0.211 ***	0.606 ***	0.018 *	0.012
	−3.85	−5.58	−2.03	−0.8
*edu_child*	0.032 ***	0.146 ***	0.306 ***	−0.119 ***
	−1.95	−2.37	−15.71	(−3.79)
Year	Controlled	Controlled	Controlled	Controlled
City	Controlled	Controlled	Controlled	Controlled
Pseudo-*R*^2^	0.139	0.126	0.088	0.046
Sample size	18,480	3381	16,206	5647

Note: The number in brackets is the *t*-value; * and *** indicate significance at the 0.1 and 0.01 levels of the estimated coefficients, respectively, and the estimated result of the constant term is omitted.

## Data Availability

The data can be made available upon request.
